# The Flavonoid Rich Black Currant (*Ribes nigrum*) Ethanolic Gemmotherapy Extract Elicits Neuroprotective Effect by Preventing Microglial Body Swelling in Hippocampus and Reduces Serum *TNF-α* Level: Pilot Study

**DOI:** 10.3390/molecules28083571

**Published:** 2023-04-19

**Authors:** Tímea Téglás, Emőke Mihok, Zoltán Cziáky, Neli-Kinga Oláh, Csaba Nyakas, Endre Máthé

**Affiliations:** 1 Research Center for Molecular Exercise Science, Hungarian University of Sports Science, H-1123 Budapest, Hungary; 2 Department of Morphology and Physiology, Faculty of Health Sciences, Semmelweis University, H-1088 Budapest, Hungary; 3 Doctoral School of Animal Science, Faculty of Agricultural and Food Sciences and Environmental Management, University of Debrecen, H-4032 Debrecen, Hungary; 4 Agricultural and Molecular Research Institute, University of Nyíregyháza, H-4400 Nyíregyháza, Hungary; 5 Faculty of Pharmacy, Vasile Goldis Western University of Arad, 310414 Arad, Romania; 6 Plantextrakt Ltd., 407059 Rădaia, Romania; 7 Institute of Nutrition Science, Faculty of Agricultural and Food Sciences and Environmental Management, University of Debrecen, H-4032 Debrecen, Hungary; 8 Institute of Life Sciences, Faculty of Medicine, Vasile Goldis Western University of Arad, 310414 Arad, Romania

**Keywords:** *Ribes nigrum*, gemmotherapy, neuroinflammation, *TNF-α*, microglia

## Abstract

Many plant-derived flavonoids are known for their anti-neuroinflammatory and anti-neurodegenerative effects. The fruits and leaves of the black currant (BC, *Ribes nigrum*) contain these phytochemicals with therapeutic benefits. The current study presents a report on a standardized BC gemmotherapy extract (BC-GTE) that is prepared from fresh buds. It provides details about the phytoconstituent profile specific to the extract as well as the associated antioxidant and anti-neuroinflammatory properties. The reported BC-GTE was found to contain approximately 133 phytonutrients, making it unique in its composition. Furthermore, this is the first report to quantify the presence of significant flavonoids such as luteolin, quercetin, apigenin, and kaempferol. *Drosophila melanogaster*-based tests revealed no cytotoxic but nutritive effects. We also demonstrated that adult male Wistar rats, pretreated with the analyzed BC-GTE and assessed after lipopolysaccharide (LPS) injection, did not show any apparent increase in body size in the microglial cells located in the hippocampal CA1 region, while in control experiments, the activation of microglia was evident. Moreover, no elevated levels of serum-specific *TNF-α* were observed under the LPS-induced neuroinflammatory condition. The analyzed BC-GTE’s specific flavonoid content, along with the experimental data based on an LPS-induced inflammatory model, suggest that it possesses anti-neuroinflammatory/neuroprotective properties. This indicates that the studied BC-GTE has the potential to be used as a GTE-based complementary therapeutic approach.

## 1. Introduction

In general terms, research on flavonoids involves the isolation, identification, characterization, and understanding of their functions, along with investigating potential health benefits associated with their consumption [1]. Flavonoids are an important class of natural phytonutrients and are well known for their protective effects against neurodegeneration and neuroinflammation [2,3]. The cytokine *TNF-α* (tumor necrosis factor alpha) is a pro-inflammatory molecule of the Th1-class and is known to play a crucial role in brain development by affecting the development and function of the hippocampus [4]. However, elevated levels of *TNF-α* can indicate the presence of inflammation. The neuroprotective effect of flavonoid-rich foods or drinks has been linked to enhanced neuronal connection and communication, including an ability to suppress neuroinflammation. These effects have the potential to promote memory, learning, and cognitive function in the hippocampus [5,6,7]. Growing evidence suggests that sustained neuroinflammation, caused by microglia activation, is implicated in the development of several neurological disorders [8]. The black currant (BC, *Ribes nigrum*) emerges as a superfood due to its phytonutrient profile and the associated health effects [9]. Some BC extracts prepared from fruits or leaves could elicit anti-inflammatory and antioxidant properties, respectively. A study on BC fruit juice had shown to lower *TNF-α* gene expression in LPS (lipopolysaccharide) induced cultured macrophages [10]. Other studies carried out on diet-induced obese mice demonstrated that a BC fruit extract reduced obesity-induced inflammation in adipose tissue and splenocytes by lowering *TNF-α* transcription [11]. Furthermore, BC fruit extract repressed obesity-associated M1 polarization of both murine and human macrophages by reducing the expression of pro-inflammatory genes such as *TNF-α* [12]. The above-mentioned studies, which mainly focused on in vitro experiments, suggested that BC extracts made from fruits or leaves had anti-inflammatory properties. However, no clear correlations were established between the biological effect and the extract-specific phytoconstituent profile, which remained largely unknown.

Therefore, we have proposed to obtain a BC-GTE that is standardized with respect to its flavonoid content and antioxidant activity, as these features would greatly facilitate the reproducibility of experiments. Next, we used a *Drosophila melanogaster*-based in vivo nutritional test system to assess the BC-GTE effect on viability. Furthermore, to investigate the anti-neuroinflammatory properties of our BC-GTE, we conducted an in vivo test using rats (*Rattus norvegicus*), where lipopolysaccharide was applied to induce inflammation through microglia activation [13]. The fact that natural flavonoids have anti-neuroinflammatory properties [14], along with the flavonoid content of the BC-GTE, provided strong justification for conducting experiments. Therefore, we examine the effect of BC-GTE pretreatment on microglia activation and *TNF-α* production during LPS-induced inflammation.

## 2. Results

### 2.1. The BC-GTE Contains Important Flavonoids and Features Relevant In Vitro Antioxidant Properties

The BC-GTE was subjected to a HPLC–ESI-MS analysis, which detected the presence of 133 phytonutrients. These phytonutrients were primarily from the flavonoid class of polyphenols, although other chemical compounds such as carboxylic acids, amino acids, vitamins, alkaloids, esters, terpenes, and others were also identified (Table 1). Our qualitative analysis of the GTE confirmed the presence of several phytonutrients that are specific to BC, including acacetin, ampelopsin, apigenin, astragalin, eriodictyol, genkwanin, isorhamnetin, narcissin, naringenin, rhamnetin, sakuranetin, tetrahydroxychalcone, galloylglucose, phloretin, phlorizin, tetrahydroxy-methoxy chalcone, and abscisic acid. This is the first time that the presence of these compounds has been confirmed through our analysis. Surprisingly, no anthocyanins could be revealed in the BC-GTE, though others have already reported their presence in BC fruits. The trigonelline and 4-hydroxy isoleucine were detected in the BC-GTE, while their incidence in the fenugreek (*Trigonella foenum-graecum*) was invoked to explain the associated antidiabetic properties [15]. Based on this similarity, it seems logical to predict an antidiabetic property for the BC-GTE, whereas this has already been confirmed for the fruits of BC [9]. Furthermore, the fact that kynurenic acid and tryptophan are present in BC-GTE suggests a potential neuroprotective function, as these compounds have been extensively studied for their implications in brain function and protection [16]. The identified components that are BC-GTE-specific suggest that its administration could produce several biological effects. It is also possible that the generated effects would have either synergistic, cumulative or even antagonistic features that could affect the viability of animal/human individuals fed/treated with BC-GTE.

**Figure 1:**
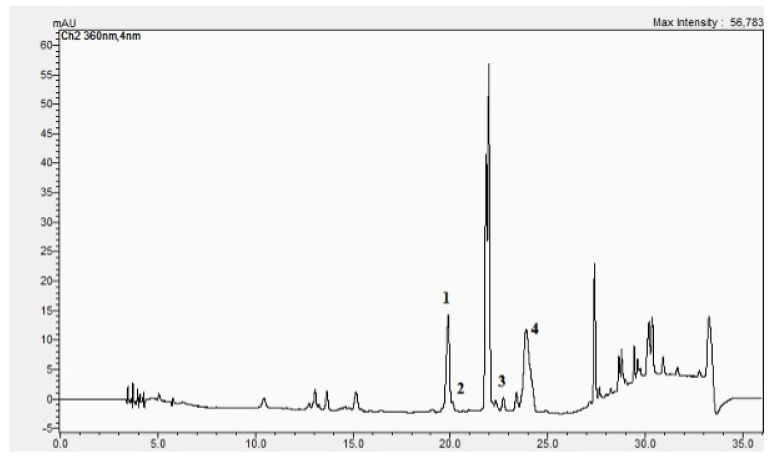
The HPLC chromatogram of BC-GTE. 1 = quercetine, 2 = luteolin, 3 = kaempferol, 4 = apigenin.

Noticeably, our study indicates that the flavonoids are the most numerous phytonutrients in the BC-GTE since we could identify about 80 of them, comprising both non-glycosidic and glycoside types. The most important non-glycosidic flavonoids are luteolin, quercetin, and apigenin, which together quantitatively represent 91.7% of all flavonoids, whereas the presence of kaempferol looks diminished (see Table 2 and Figure 1). Noteworthy, the BC-GTE showed relevant in vitro antioxidant properties as demonstrated by all four methods used (see Materials and Methods Section).

### 2.2. The BC-GTE Does Not Feature Cytotoxicity in the Case of Drosophila melanogaster

**Figure 2:**
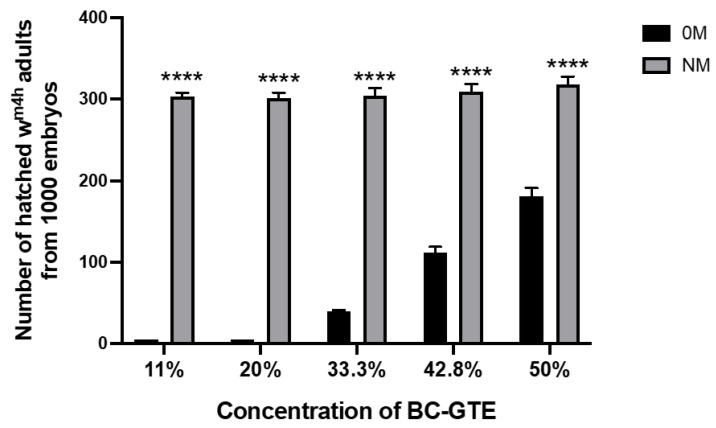
Effects of exposure to different concentrations of BC-GTE on the survival of *Drosophila melanogaster.* Abbreviations: 0M-zero media, NM-normal media; (****) *p* < 0.0001 zero media vs. normal media group. The values are mean ± SEM, n = 1000.

To assess the putative viability or cytotoxic properties of the BC-GTE, we have used a *Drosophila melanogaster*-based nutritive test system where embryos of the same age and genotype were put on 0M and NM culture conditions supplemented with the BC-GTE at different concentrations (see Materials and Methods). Therefore, by mixing 0.5 mL, 1 mL, 2 mL, 3 mL, and 4 mL of BC-GTE stock solution with 4 mL of 0M or NM culture media, we were able to assess the BC-GTE-generated effect at concentrations such as 11, 20, 33.3, 42.8, and 50%. The 0M represented a dietary condition that did not provide nutrients to support the larval development of fruit flies, and consequently, the hatched first-instar larvae would die instantly due to the lack of nutrients. Interestingly, when the 0M contained 11% and 20% of BC-GTE, no 2nd instar larvae were observed in the culture vessels, suggesting that the extract was not able to cover the nutritional requirements of the early phase of larval development (Figure 2). However, when 33.3%, 42.8%, and 50% of BC-GTE concentrations were applied in the case of the 0M condition, we could observe the adult-specific survival rates increasing from 4% to 11% and 18%, respectively. The surviving fruit fly adults showed normal body sizes, and no variegating white phenotype was visible for the wm4h strain. This suggests that the BC-GTE did not affect the expression of the white gene. Noteworthy, increasing the BC-GTE concentrations over 50% did not yield higher adult viability in combination with the 0M. Furthermore, we also assessed the effects of the above-mentioned BC-GTE concentrations among the NM type of dietary conditions. The normal medium is considered to provide the optimal (standard) nutrient composition for the larval development of fruit flies. When the NM was supplemented with the BC-GTE, it did not significantly affect the about 30% viability level of the wm4h individuals. These observations indicate that under normal dietary conditions, the BC-GTE does not have any effect on enhancing viability, but in the case of 0M dietary restrictions, it may provide some support to the development of fruit fly larvae. Taken together, we must conclude that the BC-GTE does not feature cytotoxicity and should contain relevant nutrients for larval development. It should be noted that in our evaluation of several GTEs for their nutritional properties on a 0M-based diet, we observed that all extracts containing tryptophan, an essential amino acid, were able to support the development of fruit fly larvae to adulthood to some extent, as shown in unpublished results by Máthé E. Besides the rich phytonutrient profile, the nutrition-based viability tests all indicate that the BC-GTE is a fairly complex matrix that requires thoughtful examination if the associated biological effects are to be assessed.

### 2.3. The BC-GTE Contains Many Phytonutrients with Reported Anti-Inflammatory and Neuroprotective Properties

Several previously published studies indicated that flavonoids possess important anti-inflammatory properties [14]. In the following, we will consider some of the most relevant flavonoids identified in BC-GTE with regard to their proven physiological effects. The luteolin could activate M2 and suppress M1 macrophages [17]. It suppressed the nuclear factor kappa-light-chain-enhancer of activated B cells (NF-κB) [18], p53 [19], and phosphoinositide 3-kinases/protein kinase B (PI3K/Akt) [20] signaling pathways in inflammation-associated pathological conditions. The positive implication of quercetin in regulating macrophage activation and M1/M2 polarization was revealed [21,22], while the LPS counteracting anti-inflammatory effect was also identified [23,24]. Apigenin was found to have anti-inflammatory properties when microglia were stimulated by LPS, as it induced the glycogen synthase kinase-3 beta/nuclear factor erythroid 2–related factor 2 (GSK3β/Nrf2) signaling pathway [25] and modulated the expression of enzymes involved in the tryptophan/kyneurin pathway [26]. Kaempferol was shown to exert neuroprotective [27] and anti-neuroinflammatory [28] properties, and it would reduce the interleukin-1β (IL-1β)-induced inflammation by suppressing the NF-κB pathway [29]. Rutin and naringenin are two flavonoids identified at low concentrations in BC-GTE. Rutin, which is a glycoside of the flavonoid quercetin, was shown to have a strong protective effect in the case of neurodegenerative diseases [30], and it is also appreciated for its anticancer, anti-diabetic, and antimicrobial properties [31]. The compound naringenin had protective effects in the case of liver diseases [32]. Additionally, it has demonstrated neuroprotective [33] and anti-diabetic [34] properties.

Further to flavonoids, another numerous category of phytonutrients seen in the BC-GTE is the one with different carboxylic acids (CA). Interestingly, the most extensively studied compounds in this group have been found to possess anti-inflammatory properties, such as caffeic acid, which has been shown to be relevant for intestinal inflammation [35] and metabolic syndrome [36]. Other CAs found in BC-GTE were the ferulic acid, with anti-inflammatory and neuroprotective roles [37], while the jasmonic acid, showing structural similarity to prostaglandins, was able to inhibit inflammation through several mechanisms [38]. Chlorogenic acid was also identified in the BC-GTE, and its neuroprotective role was associated with anti-oxidant and anti-inflammatory mechanisms [39]. It has been demonstrated that, in combination with other plant-derived bioactive compounds such as epigallocatechin-3-O-gallate, resveratrol, and curcumin, it could alleviate neurodegenerative diseases [40]. Among the BC-GTE identified Cas, we should also mention that α-linolenic acid, which is an omega-3 type of fatty acid, has many experimentally proven health benefits, including neuroprotection [41].

It is essential to notice that the multiple anti-inflammatory effects reported in the case of individual flavonoids and carboxylic acids have not been thoroughly reviewed. Nevertheless, the presence and combination of these phytonutrients strongly indicate that the BC-GTE might possess neuroprotective and/or anti-inflammatory properties.

### 2.4. The BC-GTE Pretreatment Overcomes the Swelling of Rat Microglial Cell Body, following LPS Activation

After observing the absence of cytotoxicity in vivo through *Drosophila melanogaster*-based tests, as well as the significant flavonoid content and relevant in vitro antioxidant activity, we decided to investigate the anti-inflammatory potential of the BC-GTE. For this purpose, we utilized the rat (*Rattus norvegicus*)-specific LPS-induced neuroinflammatory model, which is widely accepted in the field of inflammation research (for review, see [42]). Firstly, in this pilot study, we aimed to measure the acute response of the peripheral immune system to LPS administration in rats. It was previously described that the concentrations of cytokines and chemokines markedly increased after 3 h via 1 mg/kg body weight LPS administration [43]. Our second aim was to study microglia activation in the rat brain, and we decided on the 72 h time point after LPS injection. It has already been demonstrated that mice infected with live *E. coli* showed microglial activation 72 h post-inoculation, with increased cell number in the cortex, hippocampus, and thalamus as compared to controls. At 72 h, flow cytometry of microglia from *E. coli*-infected mice showed increased cell size [13,44]. Interestingly, in many rodent-based experiments, peripheral inflammatory stimuli such as the LPS could activate microglial cells and increase the *TNF-α* level in the brain. These are features that are specific for neuroinflammation [13,45]. It is well documented that activated microglial cells play a determinative role in brain inflammation, leading to the swelling of the microglial cell body, thickening of proximal processes, and reduction in distal ramifications [46]. These phenomena can be seen in the case of aging and neurodegenerating brains too [47,48].

**Figure 3:**
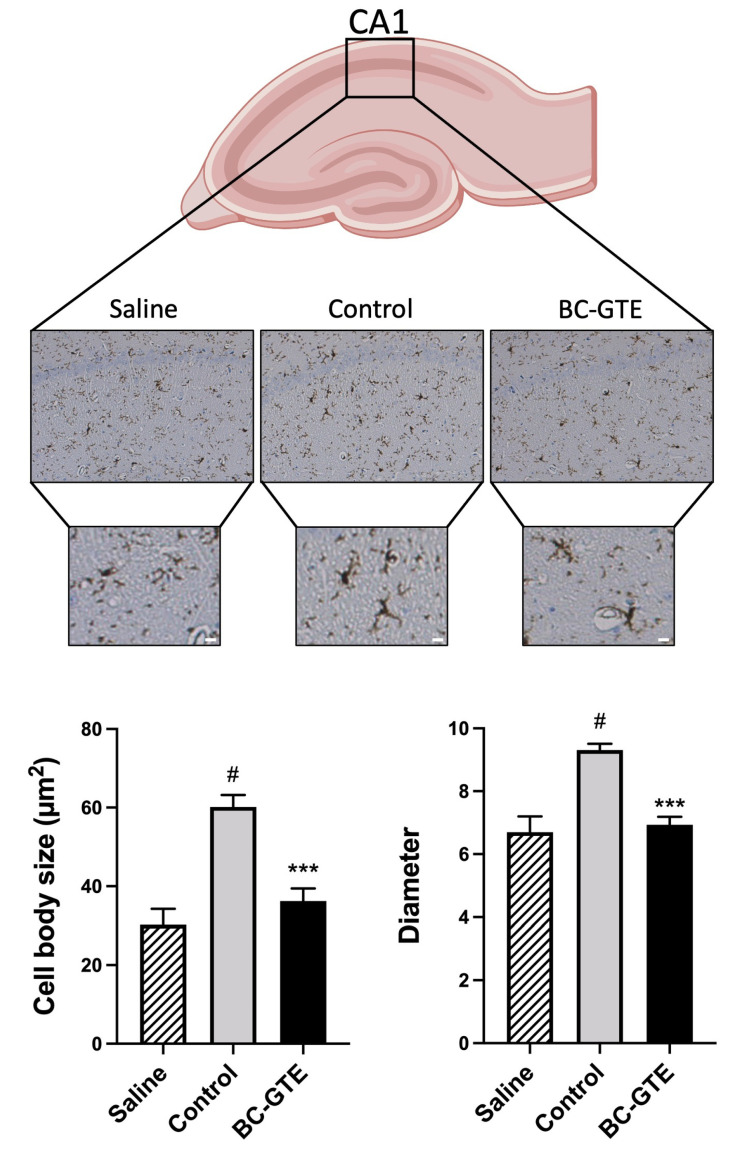
Experimental data presenting the physiological effects of BC-GTE on LPS-induced adult rats. The microglial cell body swelling (B, C). Abbreviations: BC-GTE—extract treated animal group; (***) *p* < 0.001 control vs. BC-GTE group; (^#^) *p* < 0.01 control vs. saline group. The values are mean ± SEM, n = 30–60. Scale bar: 10 µm.

Having in mind that we were able to reproduce the above-described neuro-inflammatory scenario, we designed another experimental setup where, before the LPS injection, the rats received ad libitum water with BC-GTE (see Materials and Methods). Analyzing the hippocampal CA1 area of the rats 72 h after LPS injection, we observed a significant reduction in the body size of microglia in the case of BC-GTE pretreatments compared to controls (*p* < 0.001; Figure 3). The LPS-injected control animals had significantly greater body sizes compared to the non-LPS-induced saline group (*p* < 0.01), an effect that indicates the inflammatory potential of the LPS. Moreover, no significant differences are apparent among all samples regarding the microglia number and the thickening of the proximal processes of microglial cells in all analyzed samples (Figure 3). Therefore, our observations are suggesting that the pre-treatment with BC-GTE could efficiently prevent/suppress the microglial activation-induced swelling of the cell body in the hippocampus upon the LPS-induced neuroinflammation.

### 2.5. The BC-GTE Pretreatment Leads to Diminished Serum TNF-α Level in the Serum

**Figure 4:**
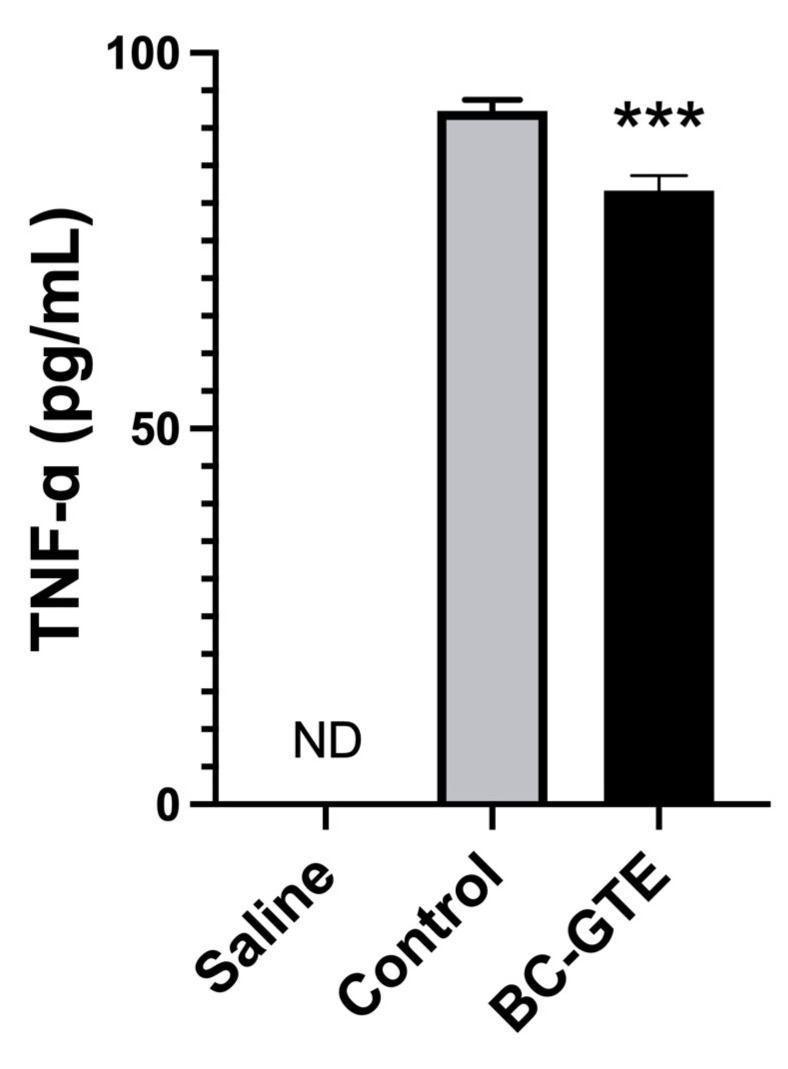
Experimental data presenting the serum *TNF-α* levels of BC-GTE on LPS-induced adult rats. Abbreviations: BC-GTE—extract treated animal group; ND—not detected. (***) *p* < 0.001 control vs. BC-GTE group. The values are mean ± SEM, n = 6.

As mentioned earlier, luteolin, quercetin, apigenin, and kaempferol, the most important flavonoids identified in BC-GTE when assessed independently, were reported to have immunomodulatory and anti-inflammatory properties. Other studies indicated that the stimulation of the innate immune system through LPS injection could activate microglial cells. Their morphological changes would be accompanied by secreting pro-inflammatory cytokines such as *TNF-α* [13], eventually leading to neuroinflammation [45]. To further substantiate the anti-inflammatory properties of the BC-GTE, we analyzed the level of *TNF-α* in rat serum-specific after LPS injection. We observed that in the BC-GTE pretreated animals, the serum-specific concentration of *TNF-α* appeared significantly diminished compared to the control animals or the BC-GTE non-pretreated control individuals (Figure 4). Moreover, we could not detect *TNF-α* in three types of samples: (i) the “saline” group, where animals were pretreated with the solvent mix of the BC-GTE and the LPS was replaced with the saline injection; (ii) the control animals before LPS injection; and (iii) the animals pretreated with BC-GTE before LPS injection. Our results indicated that the pretreatment with BC-GTE prevented the rise of the circulating *TNF-α* levels following LPS injection, further suggesting that the BC-GTE would possess a relevant anti-neuroinflammatory effect.

## 3. Materials and Methods

### 3.1. Preparation of BC-GTE

Fresh buds of BC were harvested annually around the February-March period in Cluj, Romania, from an organic crop culture (ECOINSPECT certificate Ro-008) for three consecutive years. Each time the extractions were performed at cold temperatures by periodic mixing of the fresh vegetal material with the solvent (96% vol. ethanol—100% glycerol = 1:1) for 20 days. The extraction ratio was 1:20, while the extracted solutions were separated from the vegetal rests by decantation and pressing at 400 atm. The obtained BC-GTEs could be stored at room temperature for at least 2 years without affecting their total flavonoid content and antioxidant activity.

### 3.2. UHPLC–ESI-MS Analysis of BC-GTE

A Dionex Ultimate 3000RS UHPLC (ultra high-performance liquid chromatography) system equipped with a Thermo Accucore C18 column, 100/2.1 with a particle size of 2.6 μm, was coupled to a Thermo Q Exactive Orbitrap mass spectrometer equipped with an electrospray ionization source (ESI), and the measurement accuracy was within 5 ppm.

### 3.3. Phytochemical Analysis of BC-GTE

The total flavonoid content (TFC) was evaluated by the spectrophotometric method as described in the Romanian Pharmacopoeia (1993) at 430 nm with AlCl_3_ as a coloring agent. The flavonoids were determined quantitatively using a Shimadzu Nexera-i HPLC equipped with Fortis C18 columns (150 × 2.1 mm × 3 µm) and a UV–Vis DAD detector at 360 nm. The elution was performed with a solvent gradient (see Supplementary Table S1). The standards for the identification of apigenin, kaempferol, luteolin, and quercetin were obtained from Phytolab (Germany), while calibration curves were required for their quantitation (see Table 3). The antioxidant capacity of the BC-GTE was evaluated using methods such as FRAP (Ferric reducing antioxidant power), CUPRAC (cupric reducing antioxidant capacity), superoxide radical, and xanthinoxidase inhibition [49,50,51]. FRAP and CUPRAC are spectral methods to evaluate the antioxidant capacity. FRAP is the ferric-reducing ability of plasma, while CUPRAC is the cupric ion-reducing antioxidant capacity. All assessments were performed in technical triplicates.

### 3.4. Cytotoxicity Studies on Drosophila Melanogaster

To assess the putative cytotoxicity of BC-GTE, *Drosophila melanogaster*-based nutrition tests were carried out using the wm4h (white mottled 4) mutant strain. These experiments were performed in parallel at 25 °C and by applying two different dietary conditions: [1] zero medium (0M) and [2] normal medium (NM). For this reason, synchronized 0–2 h old embryos were collected and placed on the two types of dietary setups, while the survival of embryos to adulthood was monitored. The number of adults was scored daily until no hatched adults were found. Meanwhile, the BC-GTE was assessed at 5 different concentrations: 11%, 20%, 33.3%, 42.8%, and 50% (by mixing 0.5 mL, 1 mL, 2 mL, 3 mL, and 4 mL of the GTE stock solution with 4 mL of both 0M and NM culture media). It is important to pinpoint that the wm4h individuals tested had the same genotype and age, and the experiments were conducted in triplicates. Thus, approximately 1000 embryos were examined for each evaluated BC-GTE concentration, ensuring that the results obtained were fully comparable.

### 3.5. Rattus Norvegicus Based Experimental Design

Eight months old, male Wistar rats were housed in groups (2 animals/cage) so that a standard laboratory diet and water were provided ad libitum at a 12 h light/dark cycle. The animals were randomly divided into three groups, of which two were the saline group (n = 6), the control group (C, n = 6), and the BC-GTE group (n = 6). The saline group and the control group received a diluted stock solution that contained 40% ethanol, 40% vegetable glycerin, and 20% water. The third group (BC-GTE) received a specific drink: a diluted stock solution of BC-GTE having 40% ethanol, 40% vegetable glycerin, and 20% BC bud material extract for 4 weeks. The contents of the drinking vessels were replaced daily with 250 mL of freshly prepared drinks, while the stock solutions were diluted at 1:7500 based on the recommendations of the gemmotherapy literature [52].

All experimental procedures on animals were approved by the Animal Examination Ethics Council of the Animal Protection Advisory Board at Semmelweis University (Budapest, Hungary), which fully complied with the principles of EU Directive 2010/63/EU for animal experiments.

### 3.6. The LPS-Induced Inflammatory Reaction in Rats

After a four-week long administration period, there were collected blood samples from the right foot saphenous vein. During the following day, the two groups of animals (Control and BC-GTE) were administered an intraperitoneal injection of LPS (lipopolysaccharide from Escherichia coli, Sigma-Aldrich Co., St. Louis, MO, USA) at a dose of 1 mg/kg body weight. This concentration has been used in most inflammation-related animal studies [43,53,54]. The third group was injected with saline. Three hours after injection, blood samples were collected from the left foot saphenous vein and centrifuged at 1500× *g* for 15 min at 4 °C. Following the supernatant removal, the samples were kept on ice until they were assayed.

### 3.7. Quantification of the Serum Specific *TNF-α* Levels

The concentrations of *TNF-α* were measured by a commercial *TNF-α* ELISA kit, and the protocol provided by the vendor was followed (#KRC3011, Invitrogen Co., Carlsbad, CA, USA). We measured the optical density (OD) in each sample with a microplate reader, and we calculated the mean OD from the triplicates. The OD of the sample was compared to a standard curve according to the manufacturer’s instructions. The inter- and intra-assay precisions were <10% (% coefficient of variation).

### 3.8. Immunohistochemistry Analysis

We sacrificed the animals by transcardial perfusion with saline containing heparin under CO_2_ anesthesia after 72 h of the LPS injection. The obtained brain samples were processed for immunohistochemical evaluation and stained with the IBA-1 antibody as described in [45]. Briefly, immersion-fixed 30 μm sections were pretreated with 0.3% H_2_O_2_ and incubated with 1:2500 rabbit-anti IBA-1 (#019-19741, Wako Chemicals GmbH, Neuss, Germany) in 2% BSA and 0.1% TX overnight at 4 °C. On the next day, we continued the protocol with 3 × 10 min PBS washing. We used 1:500 goat anti-rabbit secondary antibody (#8114S, Cell Signaling, Boston, MA, USA) for two hours at room temperature and avidin–biotin peroxidase complex (#PK-4004, Vectastain ABC Kit, Vector, Burlingame, CA, USA) for one hour. Sections were DAB-labeled (0.075 mg/mL DAB).

### 3.9. Analysis of Microglial Activation

Two immunohistochemically labeled pictures per rat were obtained from the hippocampal region-specific CA1 area (20× and 200×, Leica Application Suite V4.12). In each picture, a square was drawn, covering the equivalent of 50.000 µm^2^ in the case of the original microscopic section, and from 5 to 10 microglial cells were analyzed in each square. The ramified microglia can transform into an “activated state”, characterized by swollen ramified cells with a larger cell body and shorter, thicker processes, or alternatively, the microglia can adopt a “reactive state”, typically characterized by small, spherical cells. These morphological features of the immunostained microglia were assessed by image analysis software (Fiji, National Institute of Mental Health, Bethesda, MD, USA).

### 3.10. Statistical Analysis

The Statistica 13.5.0.17 soft was applied to assess the laboratory animal experiments’ specific data. The immunohistochemistry and ELISA (enzyme-linked immunosorbent assay) results were evaluated using a Student’s *t*-test. All numerical data were represented as mean ± SEM (standard error of the mean). Differences were considered significant for *p* < 0.05.

## 4. Discussion

In this paper, we present the first analysis of a BC-based GTE obtained from fresh buds. We report the extract’s chemical composition and demonstrate its anti-neuroinflammatory/neuroprotective effect. In addition to its significant antioxidant activity, the studied BC-GTE contains about 133 phytoconstituents, of which the flavonoids are the most numerous category, with more than 80 representatives, outnumbering all the other assessed bioactive compound categories. We also show that quantitatively, the BC-GTE flavonoid profile excels in luteolin, quercetin, apigenin, and kaempferol, but the presence of rutin and naringenin is also noticeable. The interference of flavonoids with inflammation and the immune system has gained much attention in the past decade [55]. For the relevant flavonoids seen in BC-GTE and when assessed individually, different types of anti-inflammatory properties could be observed [56,57,58].

Our study aims to shed light on the possible implication of the BC-GTE in overcoming neuroinflammation because the flavonoids seen in our extract were revealed to generate anti-neuroinflammatory properties [14]. Neuroinflammation should be envisioned as a central nervous system-related immune response that is triggered by injuries, PAMPs (pathogen-associated molecular patterns), or DAMPs (damage-associated molecular patterns), and conditions such as hypoxia [59,60]. After initiation, neuroinflammation can progress to the chronic phase through various factors and mechanisms. Microglial activation and the subsequent production of chemokines and cytokines are involved in the recruitment of further innate and adaptive immune cells to the inflammatory site(s) [61]. If the initial phase of a brain injury is not resolved, the above-mentioned scenario continues since no suppression/inhibition occurs. Following the establishment of chronic neuroinflammation, the aggravation of brain-related pathological conditions facilitates the irreversible progression of neurodegenerative diseases and psycho-affective disorders. According to our current knowledge, the microglia are the principal innate immune cells distributed all over the brain and regulate brain homeostasis, including all aspects of neuroinflammation, starting from the initial injury responses and ultimately protecting against PAMPs and DAMPs [62]. During the initiation of neuroinflammation, microglia are activated, reaching a pro-inflammatory status through different cellular mechanisms. This is accompanied by relevant morphological and secretory changes, which are meant to restore brain homeostasis but could also be considered markers indicating the progression of neuroinflammation. It is well known that flavonoids such as luteolin [63], quercetin [21,22], apigenin [25,26], kaempferol [28], rutin [63], and naringenin [64] have been reported to inhibit the microglia activation, which plays a crucial role in neuroinflammation. The presence of these flavonoids in the BC-GTE, we decided to assess the anti-neuroinflammatory effect of the BC-GTE led to an assessment of their anti-neuroinflammatory effect upon LPS induction. Several already reported mice- and rat-based experiments clearly demonstrated that the peripheral inflammatory LPS stimuli would cause brain-specific microglial activation associated with an increased serum-specific *TNF-α* protein level [13]. It has been demonstrated that under in vitro conditions, microglia but not astrocytes produce *TNF-α* due to LPS induction. This means that the *TNF-α* level could be considered indicative of microglial activation and neuroinflammation [65].

The above-mentioned experimental approach successfully recapitulates neuroinflammation in laboratory conditions. Therefore, it could serve as a valuable tool for defining novel therapeutic strategies to treat/prevent neurodegenerative diseases and/or psycho-affective disorders with inflammatory components [42]. Our reported experiments indicated that the BC-GTE administered before LPS injection would prevent to some extent the transformation of hippocampal microglia, which is further corroborated by the reduced serum specific *TNF-α* level. Therefore, our results suggested that the BC-GTE, through its phytoconstituent profile, could overcome the early phase of neuroinflammation, probably by not prolonging the pro-inflammatory status of microglia. It is also possible that other compounds of the BC-GTE, such as tryptophan and kynurenic acid, the flavonoid naringenin [66], or carboxylic acids such as caffeic, ferrulic, jasmonic, chlorogenic, and α-linoleic acids, might induce further neuroprotective mechanisms that would mitigate the progression of neuroinflammation.

## 5. Conclusions

Our research was based on a standardized BC-GTE whose phytonutrient profile was thoroughly analyzed both qualitatively and quantitatively. This approach could provide much-needed consistency and continuity for future studies. In addition to flavonoids, the BC-GTE contained other polyphenols, carboxylic acids, and essential amino acids and featured nutritive properties as revealed by *Drosophila melanogaster* tests. The administration of BC-GTE prior to the LPS-induced neuroinflammation appeared to decrease microglia activation in the adult rat brains and the serum-specific *TNF-α* levels. These latter results are promising but, based more on a pilot study, would require in-depth analyses regarding the elicited anti-neuroinflammatory effect. Though further studies are needed to define the cellular mechanisms behind the generated biological effects, the BC-GTE-based approach holds the promise of complex therapy in the case of damaged brain functions and related diseases.

## Figures and Tables

**Table 1:** The phytonutrient profile of BC-GTE.

Chemical Classification	Bioactive Compounds
Alkaloids	Kynurenic acid
	Trigonelline
Amino Acids	4-Guanidinobutyric acid
	4-Hydroxyisoleucine
	Asparagine
	Glutamic acid
	Isoleucine or Leucine
	Phenylalanine
	Proline
	Threonine
	Tryptophan
Others	Hydroxybenzaldehyde
	Indole-4-carbaldehyde
Esters	Ethyl gallate
	Sinapoyl-methoxyphenol
Flavonoids	Acacetin
	Ampelopsin (Ampeloptin, Dihydromyricetin)
	Apigenin
	Astragalin (Kaempferol-3-*O*-glucoside)
	Catechin
	Chrysoeriol
	Dihydrokaempferol (Aromadendrin, Katuranin)
	Dihydroxy-dimethoxyflavone isomer 1
	Flavonoids (Continue)Dihydroxy-dimethoxyflavone isomer 2
Flavonoids (Continue)	Dihydroxy-dimethoxyisoflavan
	Dihydroxy-methoxyflavanone-*O*-hexoside isomer 1
	Dihydroxy-methoxyflavanone-*O*-hexoside isomer 2
	Dihydroxy-trimethoxyflavone isomer 1
	Dihydroxy-trimethoxyflavone isomer 2
	Epicatechin
	Epigallocatechin
	Eriodictyol
	Genkwanin (Apigenin-7-*O*-methyl ether)
	Hydroxy-dimethoxyflavone
	Hydroxy-tetramethoxyflavone (Retusin)
	Hydroxy-trimethoxyflavone (Salvigenin)
	Hyperoside (Quercetin-3-*O*-galactoside, Hyperin)
	Isoquercitrin (Hirsutrin, Quercetin-3-*O*-glucoside)
	Isorhamnetin
	Isorhamnetin-3-*O*-glucoside
	Isorhamnetin-3-*O*-rutinoside (Narcissin)
	Isorhamnetin-*O*-hexoside isomer 1
	Isorhamnetin-*O*-hexoside isomer 2
	Isorhamnetin-*O*-hexoside-*O*-pentoside
	Kaempferol
	Kaempferol-3-*O*-rutinoside (Nicotiflorin)
	Kaempferol-*O*-hexoside
	Kaempferol-*O*-hexoside-di-*O*-deoxyhexoside
	Kaempferol-*O*-hexoside-*O*-pentoside-*O*-deoxyhexoside
	Kaempferol-*O*-pentoside
	Luteolin
	MyricetiFlavonoids (Continue)n
Flavonoids (Continue)	Myricetin-3′-*O*-glucoside (Cannabiscitrin)
	Myricetin-3-*O*-rutinoside
	Myricetin-*O*-(malonyl)glucoside
	Myricetin-*O*-arabinoside
	Myricetin-*O*-xyloside
	Naringenin
	Naringenin-6,8-di-*C*-glucoside
	Pentahydroxyflavanone
	Pentahydroxyflavanone isomer 1
	Pentahydroxyflavanone isomer 2
	Pentahydroxy-methoxyflavon-*O*-hexoside isomer 1
	Pentahydroxy-methoxyflavon-*O*-hexoside isomer 2
	Pentahydroxy-methoxyflavon-*O*-rutinoside
	Prodelphinidin B
	Prodelphinidin *C* isomer 1
	Prodelphinidin *C* isomer 2
	Prodelphinidin *C* isomer 3
	Prunin (Naringenin 7-*O*-glucoside)
	Quercetin
	Quercetin-3-*O*-rutinoside-7-*O*-glucoside
	Quercetin-di-*O*-hexoside
	Quercetin-*O*-(acetyl)hexoside
	Quercetin-*O*-(coumaroyl)hexoside
	Quercetin-*O*-hexoside-di-*O*-deoxyhexoside
	Quercetin-*O*-hexoside-*O*-pentoside-*O*-deoxyhexoside
	Quercetin-*O*-pentoside isomer 1
	Quercetin-*O*-pentoside isomer 2
	Rutin (Quercetin-3-*O*-rutinoside)
	Sakuranetin (4′,5-Dihydroxy-7-methoxyflavanone)
	Taxifolin (Dihydroquercetin)
	Tetrahydroxychalcone (Butein)
	Tetrahydroxy-dimethoxyflavone isomer 1
	Tetrahydroxy-dimethoxyflavone isomer 2
	Tetrahydroxy-dimethoxyflavone-*O*-hexoside isomer 1
	Tetrahydroxy-dimethoxyflavone-*O*-hexoside isomer 2
	Tetrahydroxy-dimethoxyflavone-*O*-hexoside-O-deoxyhexoside
	Tetrahydroxyflavanone-*O*-hexoside
	Tetrahydroxyflavone-*O*-(pentosyl)hexoside
	Trihydroxy-dimethoxyflavone
	Trihydroxy-methoxyflavone
	Trihydroxy-trimethoxyflavone-*O*-hexoside
	Trihydroxy-trimethoxyflavone-*O*-hexoside isomer 1
	Trihydroxy-trimethoxyflavone-*O*-hexoside isomer 2
**Carboxylic Acids**	Caffeic acid
	cis-Aconitic acid
	Dihydroxy-methoxybenzoic acid isomer 1
	Dihydroxy-methoxybenzoic acid isomer 2
	Ferulic acid
	Hydroxyhexadecanoic acid (hydroxypalmitic acid)
	Jasmonic acid
	trans-Aconitic acid
	Tuberonic acid
	α-Linolenic acid
	5-*O*-p-Coumaroylnigrumin
	Carboxylic Acids (Continue)Caffeoylglucose isomer 1
**Carboxylic Acids** (Continue)	Caffeoylglucose isomer 2
	Caffeoylglucose isomer 3
	Chlorogenic acid (3-*O*-Caffeoylquinic acid)
	Chryptochlorogenic acid (4-*O*-Caffeoylquinic acid)
	Coumaroyl-glucose isomer 1
	Coumaroyl-glucose isomer 2
	Coumaroyl-glucose isomer 3
	Coumaroylquinic acid isomer 1
	Coumaroylquinic acid isomer 2
	Coumaroylquinic acid isomer 3
	Feruloylquinic acid isomer 1
	Feruloylquinic acid isomer 2
	Feruloylquinic acid isomer 3
	Galloylglucose isomer 1
	Galloylglucose isomer 2
	Neochlorogenic acid (5-*O*-Caffeoylquinic acid)
	Phloretin
	Phlorizin (Phloridzin)
	Tetrahydroxy-methoxy chalcone
	Tuberonic acid glucoside
**Terpenes**	Abscisic acid (ABA)
	Geranylgeraniol
**Vitamins**	Adenine (B4)
	Nicotinic acid (Niacin, B3)
	Pyridoxine (B6)
	Riboflavin (B2)

**Table 2:** BC-GTE flavonoid content and antioxidant activity.

BC-GTE Flavonoids Content					BC-GTE Antioxidant Capacity			
Total Flavonoids [mg/mL]	Apigenin [mg/mL]	Kaempferol [µg/mL]	Luteolin [mg/mL]	Quercetin [mg/mL]	FRAP ^1^	CUPRAC ^1^	Superoxid Radical Inhibition ^2^	Xanthin-Oxidase Inhibition ^3^
0.82 ± 0.013	0.152 ± 0.002	3.9 ± 0.04	0.34 ± 0.003	0.26 ± 0.003	672 ± 7.1	4565 ± 48.8	4512 ± 49.6	41 ± 0.4
	18.5%		41.5%	31.7%				

^1^ mM TE/100 mL extract. ^2^ µM TE/100 mL extract. ^3^ mg AE/mL extract.

**Table 3:** Calibration curves data.

Standard	Concentration ^1^	Calibration Curve Equation ^2^	Correlation Factor	Detection Limit ^1^	Quantification Limit ^1^
Apigenin	50–360	42,007 × c[µg/mL] – 218,952	0.9990	15.6	26.1
Kaempferol	40–300	38,007 × c[µg/mL] + 772,543	0.9799	40.6	81.3
Luteolin	40–300	32,470 × c[µg/mL] + 441,347	0.9945	27.2	54.4
Quercetin	50–400	10,272 × c[µg/mL] + 17,725	0.9926	3.5	6.9

^1^ µg/mL. ^2^ area.

## Data Availability

The data are available from the authors upon reasonable request.

## Supplementary Materials

The following supporting information can be downloaded at: https://www.mdpi.com/article/10.3390/molecules28083571/s1. Table S1: Solvent gradient.

## References

[1] Panche A.N., Diwan A.D., Chandra S.R. (2016). Flavonoids: An overview. J. Nutr. Sci..

[2] Calis Z., Mogulkoc R., Baltaci A.K. (2020). The Roles of Flavonols/Flavonoids in Neurodegeneration and Neuroinflammation. Mini Rev. Med. Chem..

[3] Arulselvan P., Fard M.T., Tan W.S., Gothai S., Fakurazi S., Norhaizan M.E., Kumar S.S. (2016). Role of Antioxidants and Natural Products in Inflammation. Oxid. Med. Cell. Longev..

[4] Golan H., Levav T., Mendelsohn A., Huleihel M. (2004). Involvement of Tumor Necrosis Factor Alpha in Hippocampal Development and Function. Cereb. Cortex.

[5] Spencer J.P.E. (2009). Flavonoids and brain health: Multiple effects underpinned by common mechanisms. Genes Nutr..

[6] Vauzour D., Vafeiadou K., Rodriguez-Mateos A., Rendeiro C., Spencer J.P.E. (2008). The neuroprotective potential of flavonoids: A multiplicity of effects. Genes Nutr..

[7] Rendeiro C., Spencer J.P.E., Vauzour D., Butler L.T., Ellis J.A., Williams C.M. (2009). The impact of flavonoids on spatial memory in rodents: From behaviour to underlying hippocampal mechanisms. Genes Nutr..

[8] Brás J.P., Bravo J., Freitas J., Barbosa M.A., Santos S.G., Summavielle T., Almeida M.I. (2020). TNF-alpha-induced microglia activation requires miR-342: Impact on NF-kB signaling and neurotoxicity. Cell Death Dis..

[9] Cortez R.E., Mejia E.G. (2019). de Blackcurrants (Ribes nigrum): A Review on Chemistry, Processing, and Health Benefits. J. Food Sci..

[10] Huebbe P., Giller K., de Pascual-Teresa S., Arkenau A., Adolphi B., Portius S., Arkenau C.N., Rimbach G. (2012). Effects of blackcurrant-based juice on atherosclerosis-related biomarkers in cultured macrophages and in human subjects after consumption of a high-energy meal. Br. J. Nutr..

[11] Benn T., Kim B., Park Y.K., Wegner C.J., Harness E., Nam T.G., Kim D.O., Lee J.S., Lee J.Y. (2014). Polyphenol-rich blackcurrant extract prevents inflammation in diet-induced obese mice. J. Nutr. Biochem..

[12] Lee Y., Lee J.-Y. (2019). Blackcurrant (Ribes nigrum) Extract Exerts an Anti-Inflammatory Action by Modulating Macrophage Phenotypes. Nutrients.

[13] Hoogland I.C.M., Houbolt C., van Westerloo D.J., van Gool W.A., van de Beek D. (2015). Systemic inflammation and microglial activation: Systematic review of animal experiments. J. Neuroinflamm..

[14] Chen Y., Peng F., Xing Z., Chen J., Peng C., Li D. (2022). Beneficial effects of natural flavonoids on neuroinflammation. Front. Immunol.

[15] Wadhwa G., Krishna K.V., Taliyan R., Tandon N., Yadav S.S., Banerjee D., Narwaria A., Katiyar C., Dubey S.K. (2022). A novel UPLC-MS/MS method for simultaneous quantification of trigonelline, 4-hydroxyisoleucine, and diosgenin from Trigonella foenum-graecum extract: Application to pharmacokinetic study in healthy and type 2 diabetic rats. Biomed. Chromatogr..

[16] Ostapiuk A., Urbanska E.M. (2022). Kynurenic acid in neurodegenerative disorders-unique neuroprotection or double-edged sword?. CNS Neurosci. Ther..

[17] Gong B., Zheng Y., Li J., Lei H., Liu K., Tang J., Peng Y. (2022). Luteolin activates M2 macrophages and suppresses M1 macrophages by upregulation of hsa_circ_0001326 in THP-1 derived macrophages. Bioengineered.

[18] Zhang J.X., Xing J.G., Wang L.L., Jiang H.L., Guo S.L., Liu R. (2017). Luteolin Inhibits Fibrillary β-Amyloid1-40-Induced Inflammation in a Human Blood-Brain Barrier Model by Suppressing the p38 MAPK-Mediated NF-κB Signaling Pathways. Molecules.

[19] Zhang K.K., Wang H., Qu D., Chen L.J., Wang L.B., Li J.H., Liu J.L., Xu L.L., Yoshida J.S., Xu J.T. (2021). Luteolin Alleviates Methamphetamine-Induced Hepatotoxicity by Suppressing the p53 Pathway-Mediated Apoptosis, Autophagy, and Inflammation in Rats. Front. Pharmacol..

[20] Tan X.H., Zhang K.K., Xu J.T., Qu D., Chen L.J., Li J.H., Wang Q., Wang H.J., Xie X.L. (2020). Luteolin alleviates methamphetamine-induced neurotoxicity by suppressing PI3K/Akt pathway-modulated apoptosis and autophagy in rats. Food Chem. Toxicol..

[21] Tsai C.F., Chen G.W., Chen Y.C., Shen C.K., Lu D.Y., Yang L.Y., Chen J.H., Yeh W.L. (2021). Regulatory Effects of Quercetin on M1/M2 Macrophage Polarization and Oxidative/Antioxidative Balance. Nutrients.

[22] Fan H., Tang H.B., Shan L.Q., Liu S.C., Huang D.G., Chen X., Chen Z., Yang M., Yin X.H., Yang H. (2019). Quercetin prevents necroptosis of oligodendrocytes by inhibiting macrophages/microglia polarization to M1 phenotype after spinal cord injury in rats. J. Neuroinflamm..

[23] Xiong G., Ji W., Wang F., Zhang F., Xue P., Cheng M., Sun Y., Wang X., Zhang T. (2019). Quercetin Inhibits Inflammatory Response Induced by LPS from Porphyromonas gingivalis in Human Gingival Fibroblasts via Suppressing NF-κB Signaling Pathway. Biomed Res. Int..

[24] Cai S.Q., Zhang Q., Zhao X.H., Shi J. (2021). The In Vitro Anti-Inflammatory Activities of Galangin and Quercetin towards the LPS-Injured Rat Intestinal Epithelial (IEC-6) Cells as Affected by Heat Treatment. Molecules.

[25] Chen P., Huo X., Liu W., Li K., Sun Z., Tian J. (2020). Apigenin exhibits anti-inflammatory effects in LPS-stimulated BV2 microglia through activating GSK3β/Nrf2 signaling pathway. Immunopharmacol. Immunotoxicol..

[26] Kurniati D., Hirai S., Egashira Y. (2022). Effect of apigenin on tryptophan metabolic key enzymes expression in lipopolysaccharide-induced microglial cells and its mechanism. Heliyon.

[27] Chang S., Li X., Zheng Y., Shi H., Zhang D., Jing B., Chen Z., Qian G., Zhao G. (2022). Kaempferol exerts a neuroprotective effect to reduce neuropathic pain through TLR4/NF-ĸB signaling pathway. Phytother. Res..

[28] Park S.E., Sapkota K., Kim S., Kim H., Kim S.J. (2011). Kaempferol acts through mitogen-activated protein kinases and protein kinase B/AKT to elicit protection in a model of neuroinflammation in BV2 microglial cells. Br. J. Pharmacol..

[29] Zhuang Z., Ye G., Huang B. (2017). Kaempferol Alleviates the Interleukin-1β-Induced Inflammation in Rat Osteoarthritis Chondrocytes via Suppression of NF-κB. Med. Sci. Monit..

[30] Enogieru A.B., Haylett W., Hiss D.C., Bardien S., Ekpo O.E. (2018). Rutin as a Potent Antioxidant: Implications for Neurodegenerative Disorders. Oxid. Med. Cell. Longev..

[31] Ganeshpurkar A., Saluja A.K. (2017). The Pharmacological Potential of Rutin. Saudi Pharm. J. SPJ Off. Publ. Saudi Pharm. Soc..

[32] Hernández-Aquino E., Muriel P. (2018). Beneficial effects of naringenin in liver diseases: Molecular mechanisms. World J. Gastroenterol..

[33] Nouri Z., Fakhri S., El-Senduny F.F., Sanadgol N., Abd-Elghani G.E., Farzaei M.H., Chen J.T. (2019). On the Neuroprotective Effects of Naringenin: Pharmacological Targets, Signaling Pathways, Molecular Mechanisms, and Clinical Perspective. Biomolecules.

[34] Hartogh D.J.D., Tsiani E. (2019). Antidiabetic Properties of Naringenin: A Citrus Fruit Polyphenol. Biomolecules.

[35] Zielińska D., Zieliński H., Laparra-Llopis J.M., Szawara-Nowak D., Honke J., Giménez-Bastida J.A. (2021). Caffeic Acid Modulates Processes Associated with Intestinal Inflammation. Nutrients.

[36] Muhammad Abdul Kadar N.N., Ahmad F., Teoh S.L., Yahaya M.F. (2021). Caffeic Acid on Metabolic Syndrome: A Review. Molecules.

[37] Stompor-Gorący M., Machaczka M. (2021). Recent Advances in Biological Activity, New Formulations and Prodrugs of Ferulic Acid. Int. J. Mol. Sci..

[38] Jarocka-karpowicz I., Markowska A. (2021). Therapeutic Potential of Jasmonic Acid and Its Derivatives. Int. J. Mol. Sci..

[39] Lee T.K., Kang I.J., Kim B., Sim H.J., Kim D.W., Ahn J.H., Lee J.C., Ryoo S., Shin M.C., Cho J.H. (2020). Experimental Pretreatment with Chlorogenic Acid Prevents Transient Ischemia-Induced Cognitive Decline and Neuronal Damage in the Hippocampus through Anti-Oxidative and Anti-Inflammatory Effects. Molecules.

[40] Fukutomi R., Ohishi T., Koyama Y., Pervin M., Nakamura Y., Isemura M. (2021). Beneficial Effects of Epigallocatechin-3- O-Gallate, Chlorogenic Acid, Resveratrol, and Curcumin on Neurodegenerative Diseases. Molecules.

[41] Piermartiri T., Pan H., Figueiredo T.H., Marini A.M. (2015). α-Linolenic Acid, A Nutraceutical with Pleiotropic Properties That Targets Endogenous Neuroprotective Pathways to Protect against Organophosphate Nerve Agent-Induced Neuropathology. Molecules.

[42] Skrzypczak-Wiercioch A., Sałat K. (2022). Lipopolysaccharide-Induced Model of Neuroinflammation: Mechanisms of Action, Research Application and Future Directions for Its Use. Molecules.

[43] Wu S.Y., Wang T.F., Yu L., Jen C.J., Chuang J.I., Wu F.S., Wu C.W., Kuo Y.M. (2011). Running exercise protects the substantia nigra dopaminergic neurons against inflammation-induced degeneration via the activation of BDNF signaling pathway. Brain. Behav. Immun..

[44] Hoogland I.C.M., Westhoff D., Engelen-Lee J.Y., Melief J., Valls Serón M., Houben-Weerts J.H.M.P., Huitinga I., van Westerloo D.J., van der Poll T., van Gool W.A. (2018). Microglial Activation After Systemic Stimulation With Lipopolysaccharide and Escherichia coli. Front. Cell. Neurosci..

[45] Hovens I.B., van Leeuwen B.L., Nyakas C., Heineman E., van der Zee E.A., Schoemaker R.G. (2015). Postoperative cognitive dysfunction and microglial activation in associated brain regions in old rats. Neurobiol. Learn. Mem..

[46] Fernández-Arjona M. (2017). del M.; Grondona, J.M.; Granados-Durán, P.; Fernández-Llebrez, P.; López-Ávalos, M.D. Microglia Morphological Categorization in a Rat Model of Neuroinflammation by Hierarchical Cluster and Principal Components Analysis. Front. Cell. Neurosci..

[47] Godbout J.P., Chen J., Abraham J., Richwine A.F., Berg B.M., Kelley K.W., Johnson R.W. (2005). Exaggerated neuroinflammation and sickness behavior in aged mice following activation of the peripheral innate immune system. FASEB J..

[48] Heppner F.L., Ransohoff R.M., Becher B. (2015). Immune attack: The role of inflammation in Alzheimer disease. Nat. Rev. Neurosci..

[49] Alam M.N., Bristi N.J., Rafiquzzaman M. (2013). Review on in vivo and in vitro methods evaluation of antioxidant activity. Saudi Pharm. J..

[50] Benzie I.F.F., Strain J.J. (1999). Ferric reducing/antioxidant power assay: Direct measure of total antioxidant activity of biological fluids and modified version for simultaneous measurement of total antioxidant power and ascorbic acid concentration. Methods Enzymol..

[51] Apak R., Güçlü K., Özyürek M., Çelik S.E. (2007). Mechanism of antioxidant capacity assays and the CUPRAC (cupric ion reducing antioxidant capacity) assay. Microchim. Acta.

[52] Tetau M. Gemmotherapy: A Clinical guide; Editions Similia: 2010; ISBN 9782842510503. https://www.abebooks.com/Gemmotherapy-Clinical-guide-2010-Edition-Max/31290232315/bd.

[53] Liu L., Zhang Q., Cai Y., Sun D., He X., Wang L., Yu D., Li X., Xiong X., Xu H. (2016). Resveratrol counteracts lipopolysaccharide-induced depressivelike behaviors via enhanced hippocampal neurogenesis. Oncotarget.

[54] Lin H.Y., Huang C.C., Chang K.F. (2009). Lipopolysaccharide Preconditioning Reduces Neuroinflammation Against Hypoxic Ischemia and Provides Long-Term Outcome of Neuroprotection in Neonatal Rat. Pediatr. Res..

[55] Pérez-Cano F.J., Castell M. (2016). Flavonoids, Inflammation and Immune System. Nutrients.

[56] Di Matteo V., Esposito E. (2003). Biochemical and therapeutic effects of antioxidants in the treatment of Alzheimer’s disease, Parkinson’s disease, and amyotrophic lateral sclerosis. Curr. Drug Targets. CNS Neurol. Disord..

[57] Ullah A., Munir S., Badshah S.L., Khan N., Ghani L., Poulson B.G., Emwas A.H., Jaremko M. (2020). Important Flavonoids and Their Role as a Therapeutic Agent. Molecules.

[58] Krishnaiah D., Sarbatly R., Nithyanandam R. (2011). A review of the antioxidant potential of medicinal plant species. Food Bioprod. Process..

[59] Hambali A., Kumar J., Hashim N.F.M., Maniam S., Mehat M.Z., Cheema M.S., Mustapha M., Adenan M.I., Stanslas J., Hamid H.A. (2021). Hypoxia-Induced Neuroinflammation in Alzheimer’s Disease: Potential Neuroprotective Effects of Centella asiatica. Front. Physiol..

[60] Pranzatelli M.R. (2018). Advances in biomarker-guided therapy for pediatric- and adult-onset neuroinflammatory disorders: Targeting chemokines/cytokines. Front. Immunol..

[61] Kölliker-Frers R., Udovin L., Otero-Losada M., Kobiec T., Herrera M.I., Palacios J., Razzitte G., Capani F. (2021). Neuroinflammation: An Integrating Overview of Reactive-Neuroimmune Cell Interactions in Health and Disease. Mediators Inflamm..

[62] Deus C.M., Tavares H., Beatriz M., Mota S., Lopes C. (2022). Mitochondrial Damage-Associated Molecular Patterns Content in Extracellular Vesicles Promotes Early Inflammation in Neurodegenerative Disorders. Cells.

[63] Wang S., Cao M., Xu S., Shi J., Mao X., Yao X., Liu C. (2020). Luteolin Alters Macrophage Polarization to Inhibit Inflammation. Inflammation.

[64] Zhang B., Wei Y.Z., Wang G.Q., Li D.D., Shi J.S., Zhang F. (2019). Targeting MAPK pathways by naringenin modulates microglia M1/M2 polarization in lipopolysaccharide-stimulated cultures. Front. Cell. Neurosci..

[65] Welser-Alves J.V., Milner R. (2013). Microglia are the major source of *TNF-α* and TGF-β in postnatal glial cultures; regulation by cytokines, lipopolysaccharide, and vitronectin. Neurochem. Int..

[66] Chen C., Wei Y.Z., He X.M., Li D.D., Wang G.Q., Li J.J., Zhang F. (2019). Naringenin Produces Neuroprotection Against LPS-Induced Dopamine Neurotoxicity via the Inhibition of Microglial NLRP3 Inflammasome Activation. Front. Immunol..

