# Supplementary material for: The Flavonoid Rich Black Currant (Ribes nigrum) Ethanolic Gemmotherapy Extract Elicits Neuroprotective Effect by Preventing Microglial Body Swelling in Hippocampus and Reduces Serum TNF-α Level: Pilot Study
Source: Molecules. 2023 Apr 19;28(8):3571. doi: 10.3390/molecules28083571 (PMC10145433; doi:10.3390/molecules28083571)
Supplement: Supplementary file 1 [file molecules-28-03571-s001.zip › molecules-2354561-supplementary.pdf]

Supplementary Table S1. Solvent gradient

Time, min	% Acetonitrile	% Water	% of 0.1 % formic acid
0	10	80	10
5	30	60	10
10	50	40	10
15	70	20	10
20	80	10	10
25	70	20	10
35	30	60	10
40	10	80	10
